# Case report: A novel *CACNA1S* mutation associated with hypokalemic periodic paralysis

**DOI:** 10.3389/fneur.2023.1267426

**Published:** 2023-09-29

**Authors:** Evgenii P. Nuzhnyi, Alina S. Arestova, Alexey V. Rossokhin, Anna O. Protopopova, Nataliya Yu Abramycheva, Natalia A. Suponeva, Sergey N. Illarioshkin

**Affiliations:** Research Center of Neurology, Moscow, Russia

**Keywords:** hypokalemic periodic paralysis, HypoKPP, *CACNA1S*, mutation, molecular modeling, case report

## Abstract

**Background:**

Hypokalemic periodic paralysis (HypoKPP) is a rare neuromuscular genetic disorder causing recurrent episodes of flaccid paralysis. Most cases are associated with *CACNA1S* mutation, causing defect of calcium channel and subsequent impairment of muscle functions. Due to defined management approaches early diagnosis is crucial for promptly treatment and prevention new attacks.

**Materials and methods:**

We report a case of HypoKPP associated with previously unreported mutation in *CACNA1S* gene (p.R900M). Molecular modeling of Ca_V_1.1 was applied to evaluate its pathogenicity.

**Results:**

As a patient referred between attacks neurological status, laboratory and neurophysiological examination were unremarkable. Molecular modeling predicted that the p.R900M mutation affects the process of calcium channels activation.

**Conclusion:**

Novel *CACNA1S* mutation, associated with HypoKPP was identified. Monte-Carlo energy minimization of the Ca_V_1.1 model supported the association of this mutation with this disease.

## Introduction

Hypokalemic periodic paralysis (HypoKPP) is a rare neuromuscular disorder (channelopathy), characterized by the recurrent episodic attacks of muscle weakness lasting from minutes to several days accompanied by low serum potassium (1). The majority of HypoKPP cases are inherited and caused by mutations in skeletal muscle calcium (*CACNA1S*, up to 70%) and sodium (*SCN4A*, up to 20%) channels genes, while a small proportion remains genetically undefined (2). Acquired cases of HypoKPP are associated with thyrotoxicosis and other endocrine disorders, some may result from gastrointestinal and renal potassium loss (3).

Pathological variants in many ion channel diseases are widely distributed throughout the channel proteins, whereas those in HypoKPP are almost exclusively concentrated in the voltage sensor. Most such mutations affect arginine residues in S4 segments that contribute to voltage sensing, leading to abnormal channel functioning. However, the full spectrum of HypoPP mutations has not been defined (4).

We report a case of HypoKPP with a novel *de novo* pathogenic variant in the *CACNA1S* gene, supported by using molecular modeling approach. Currently, the generally accepted mechanism for the development of HypoKPP is the occurrence of a leak current through the voltage sensor domain (VSD) of Ca_V_1.1 and Na_V_1.4 channels with mutated arginines in the S4 helix (4–6). In this work, based on the structural analysis of the Ca_V_1.1 channels, we predicted that the newly discovered p.R900M mutation affects the mobility of the IIIS4 helix, which leads to disruption of the channel activation process. We hypothesize that this mechanism also contributes to the development of HypoKPP.

## Case description

**Figure 1:**
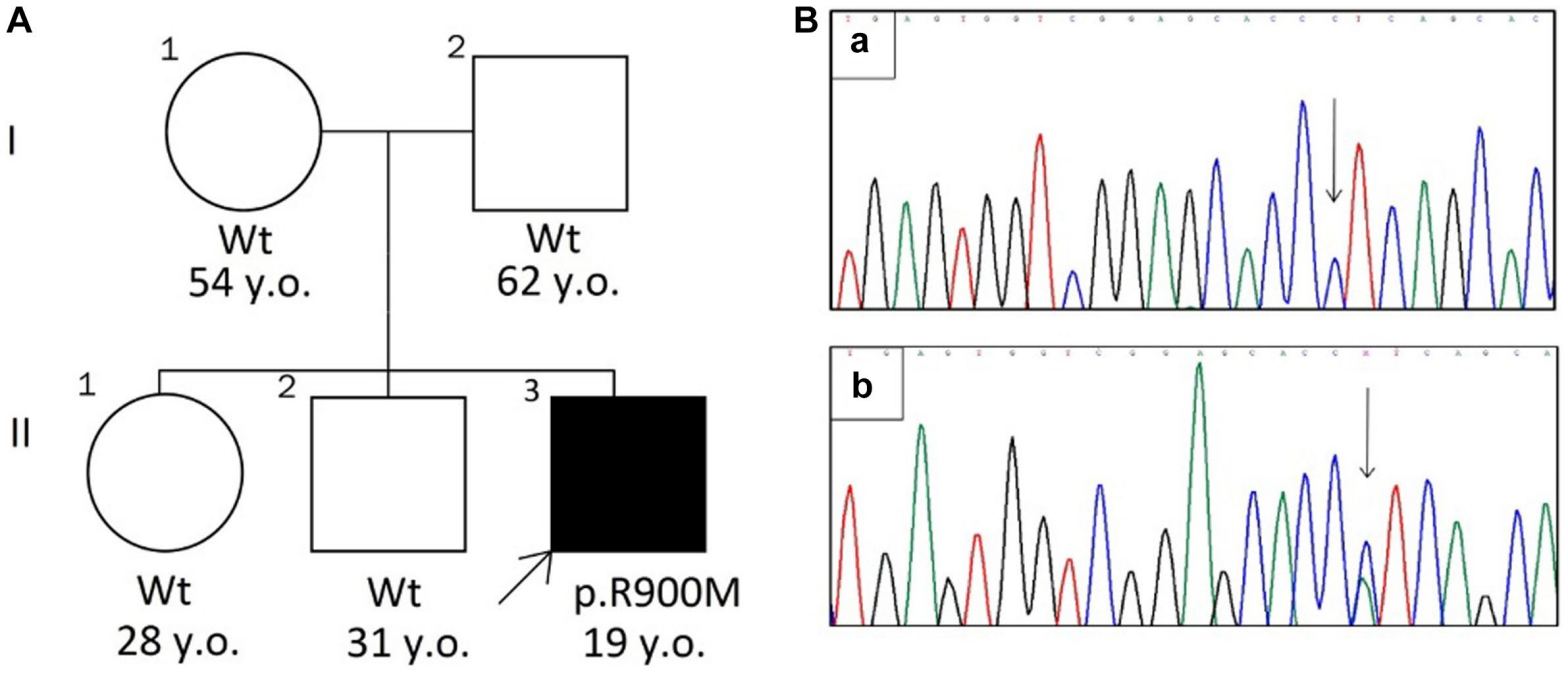
**(A)** The pedigree chart. The arrow indicates the proband with HypoKPP (Wt—wild type allele). **(B)** Sanger sequencing chromatograms (revers variant) shows wild type allele (a) and c.2699G > T mutation (b).

A 19-years old man, originated from Tajikistan, presented with severe reversible episodes of muscle weakness for 3 years. The episode starts abruptly with progressive weakness in extremities and paraspinal muscles up to complete immobility by the third day with further spontaneous recovery. The episodes reoccur 3 times a year without any noticeable triggers. Breathing, swallowing, speech, urination is always intact. Between attacks the patient feels absolutely normal and has no complaints. The medical history was unremarkable for chronic conditions, constant medication use, substance abuse. There was no family history on similar symptoms or any neurological disorders (parents, two sibs are clinically unaffected) (Figure 1A).

During interictal period on neurological examination deep tendon reflexes were diminished and slight muscle hypotonia was observed in the extremities with no signs of muscle hypotrophy, paresis or percussion myotonia.

The laboratory testing revealed normal complete blood count and comprehensive metabolic panel, including sodium and potassium levels. It should be mentioned that during the attack the potassium level has never been investigated.

Brain MRI revealed *pituitary* microadenoma 0.5 × 0.5 × 0.8 cm and blood test showed normal levels of pituitary hormones. The patient was consulted by an endocrinologist, so an underlying endocrine pathology was excluded.

Neurophysiological examination included repetitive nerve stimulation test, short exercise test according to standardized protocol and needle EMG (7). No decrement was observed on repetitive nerve stimulation test, short exercise test was also unremarkable. Prolonged exercise test was not performed. Needle EMG of m. extensor digitorum communis and m. vastus lateralis did not demonstrated any myopathic changes.

Diagnosis of primary periodic paralysis was suspected. Next-generation sequencing with a related commercial gene panel (“Neuromuscular disorders,” Illumina MiSeq) was performed. A c.2699G > T (p.Arg900Met, p.R900M, NM_000069.3, exon 21) variant in the *CACNA1S* gene was identified, satisfying the ACMG criteria of “likely pathogenic” (PS2, PM6, PM1), however according to InterVar the variant is interpreted as “uncertain significance” (8). Despite pathological variants in R900 substituting for another amino acid, like R900G and R900S, associated with HypoKPP, have already been reported (4, 9, 10), the identified variant was absent in the ExAc, gnomAD, GENOMED Databases.

The substitution was further confirmed by Sanger sequencing (Figure 1B). The mutation was not found in parents and two sibs, suggesting that it had arisen *de novo*.

In order to confirm either the novel identified variant is pathogenic and to evaluate its effect on calcium channel functioning, the molecular modeling was applied.

**Figure 2:**
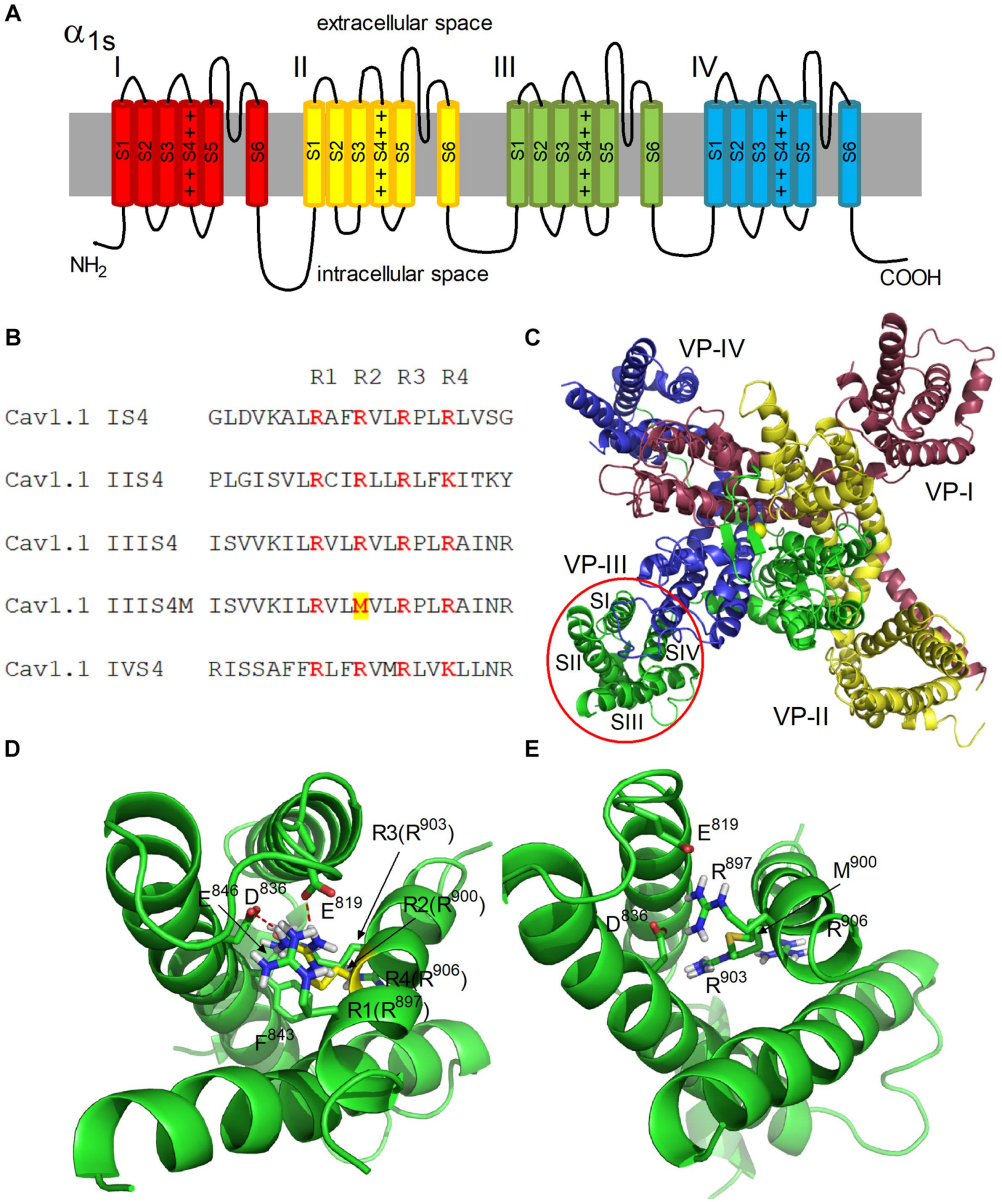
Architecture of the hetero-tetrameric voltage-gated human calcium channel Ca_V_1.1. **(A)** Schematic representation of four domains (I–IV) of the α1S subunit. S1–S6 transmembrane helices and four positive charges of S4 helix are shown in each domain. **(B)** Alignment of S4 segments of domains I-IV of Ca_V_1.1. Positively charged Arg residues (R1-R4) are highlighted in color. The Arg900Met mutation is highlighted in the IIIS4M (mutated) segment. **(C)** Structural model of the α1S subunit of Ca_V_1.1. View from the extracellular space is presented. The color coding of domains corresponds to **(A)**. Transmembrane helices S1–S4 forming a voltage sensor (*VS*) are denoted in domain III. Yellow sphere in the center represents Ca^2+^ ion in the selective filter of the channel. **(D,E)** Structural models of *VS*-III in the activated state in the native and mutated forms, respectively. Residues R1-R4 of the S4 helix are shown, as well as residues E819, D836, E846, and F843 of the S2 helix interacting with them. The side chain of R900 is highlighted in yellow **(D)**. Ionic contacts formed by R900 with residues E819 and D836 are shown as red dashed lines.

To build a homology model of the α1S subunit of the human calcium voltage-gated channel, a cryo-EM (EM - electron microscopic) structure of the inactivated Ca_V_1.1 channel (5GJW, code in the Protein Data Bank) of the rabbit, in which the voltage sensors are in the activated position, was used as a template (11). A schematic representation and structural model of the α1S subunit of Ca_V_1.1 are shown in Figures 2A,C. Model building was preceded by alignment of the amino acid sequences (UniProt entries Q13698 CAC1S_HUMAN and P07293 CAC1S_RABIT). Aligned sequences of S4 segments are shown in Figure 2B.

We used the Monte-Carlo energy minimization (MCM) method (12) to optimize the model geometry. Non-bonded interactions were calculated using the AMBER force field (13). Electrostatic interactions were calculated using the solvent exposure- and distance-dependent dielectric function (14). Energy was minimized in the space of internal model coordinates, which are the lengths of valence bonds, valence and torsion angles, Cartesian coordinates and Euler angles that determine the position of individual molecules, using the ZMM program (14).

The MC minimization was carried out in two stages. At the first stage, the energy was minimized with constraints imposed on Cα atoms. Minimizing the energy of the model with a system of constraints allows to avoid the deformation of the structure given by the template due to steric conflicts, which are inevitable in the starting geometry of the model. After a constrained MCM trajectory converged, all constraints were removed and the model was finally refined. Each MCM trajectory was terminated if 5,000 successive changes in the model coordinates did not lead to a decrease in the model energy. A detailed description of the MCM method is given in our previously published papers (15, 16).

The α1 subunit of Ca_V_ includes four domains I-IV, each of which contains six transmembrane helices S1-S6 (Figure 2A). The S5, S6 helices form a pore with a selective filter and an activation gate (17). The S1–S4 helices form the VSD of the channel (Figure 2C). Each S4 helix contains positively charged Arg and Lys residues (Figure 2B).

At the resting state, a negative membrane potential pulls the positively charged S4 helices toward the cytoplasmic side of the membrane, keeping the channel gate closed. Membrane depolarization is accompanied by a change in the electric field, which leads to a shift of S4 across the membrane plane, which initiates a conformational transition of the channel from the resting (closed) state to the open state (18, 19).

According to this mechanism, the R1–R4 charges of the S4 helix of domain III (Arg897, Arg900, Arg903, and Arg906) alternately form ion pairs with countercharges of the S2 helix (Asp836 and Glu846) when the helix moves in an electric field (20). In our Ca_V_1.1 model, *VS* is in an activated state, and Arg900 (R2) forms salt bridges with Asp836 and Glu819 residues (Figure 2D). MC minimization showed that these contacts are lost when positively charged Arg900 is replaced by nonpolar Met (Figure 2E). Thus, salt bridges between Met900 (S4) and Asp836/Glu819 (S2) in domain III cannot be formed in the mutated channel, which obviously leads to a decrease in the probability of domain III transition to an activated state. Therefore, disruption of domain III activation should decrease the probability of the entire channel transition to the open state.

Thus, our calculations predict the association of the p.R900M mutation in *CACNA1S* gene with the disease.

Since hypokalemia has not been identified in the patient, there is a chance of normokalemic periodic paralysis (NormoKPP). However, the type of mutation increases our suspicion in favor of HypoKPP and the patient was prescribed with potassium supplementation and spironolactone (25 mg per day) ex juvantibus and educated to notice and avoid possible triggers. For 10-months follow-up the patient has not experienced new attacks and the potassium level has remained normal.

## Discussion

Primary hypokalemic periodic paralysis is a rare condition, yet with established approaches to management for prevention severe attacks and life-threatening consequences (1). The majority of primary HypoKPP cases is associated with mutations in *CACNA1S* gene, which encodes the α1S subunit of the calcium voltage-gated channel Ca_V_1.1 (4). The first identified mutation in the *CACNA1S* gene was reported in 1994 (21). Up to date it has been shown that most mutations occur at positively charged arginine in the VSD (S4 helix) of the α1S subunit (4).

Activation of nicotinic acetylcholine receptors at the neuromuscular junctions leads to the entry of sodium ions and depolarization of muscle cells. Depolarization causes an influx of calcium ions through the voltage-gated Ca_V_1.1 channels and through Ca_V_1.1-bound ryanodine receptors (RYR1) in the sarcoplasmic reticulum (SR), which triggers muscle contraction. Mutation in *CACNA1S* gene (p.R900M) identified in this study impairs the functioning of the voltage sensor in domain III of the Ca_V_1.1 channels. Therefore, this mutation affects the channel opening process and results in the loss of function of these channels.

RYR1 are intracellular receptors and have no voltage-sensing structures. The Ca_V_1.1 channels associated with RYR1 act as a voltage sensor of calcium release from SR (22). Savalli et al. (23) showed that VSD III in the Ca_V_1.1 channels bound to RyR1 exhibits fast activation compatible with Ca^2+^-release kinetics form SR. Therefore, the R900M mutation may also impair calcium release from the SR through RyR1.

It was previously shown that mutations of S4 Arg residues are also accompanied by a leak current carried by protons or other monovalent cations through a gating pore (5, 6). The gating pore is a tunnel formed between S1 and S4 helices in which the S4 helix moves across the membrane in response to changes in membrane potential (24). Sokolov et al. (6) showed that mutations of the two outermost Arg669 and Arg672 to Gly in domain II of Nav1.4 result in generation of a cation leak through the gating pore at hyperpolarized potentials. In the Shaker potassium channel, mutations of the more intracellular S4 Arg to His resides resulted in a leak current carried by protons already at depolarized potentials (25). It has been suggested that such leak currents mainly contribute to the pathophysiology of HypoKPP (5, 26), supported by the fact that the muscles of HypoKPP patients are usually depolarized (27).

Here we demonstrate a HypoKPP case associated with *CACNA1S* gene mutation p.R900M, previously unreported. Matthews et al. (4) have reported a HypoKPP patient with p.R900S mutation; however, clinical characteristics were not described. Pathological variants in R900 substituting for another amino acid, p.Arg900Ser and p.Arg900Gly, have also been reported in two Chinese and Japanese families (9, 10). The clinical features of these cases and our patient are presented in Table 1.

**Table 1:** Clinical features of the cases due to R900 mutations in the *CACNA1S* gene.

Cases (references)	Family 1 (9)			Family 2 (10)			Present study
Nationality	Chinese			Japanese			Tajik
Age/Sex	25/M (sib)	27/M (sib)	46/M (father)	41/M (sib)	35/M (sib)	69/M (father)	19/M
Onset age	16	17	17	21	13	13	16
Frequency of attacks (times per year)	12–100	10–12	10–40	5	10	5	3
Duration of attack (hours)	24–48	12–24	<24	12–48	No data	No data	72–84
Attack triggers	High carbohydrate meal, exhaustion, staying up late, prolonged immobility	High carbohydrate meal, exhaustion	Exhaustion, high carbohydrate meal, alcohol, coldness	Hard physical exercises	Hard physical exercises, overeating		Not identified
Mutation	c.2700G > C (p.R900S)			c.2698A > G (p.R900G)			c.2699G > T (p.R900M)

All patients had a typical HypoPP phenotype with attacks onset in the second decade. Also all patients were male, which may confirm the hypothesis of different gender penetrance and disease severity in males and females (28). In our patient, the frequency of attacks was less, but the attacks were more severe (up to 84 h). We did not identify any attack triggers; however, this fact does not contradict the diagnosis. It can be assumed that the R900 mutations in the *CACNA1S* gene are associated with the classical phenotype of HypoKPP with varying severity.

In rare cases, mutations in the *CACNA1S* gene can cause Ca_V_1.1-related myopathy (congenital and late-onset limb-girdle forms) with both autosomal dominant and recessive inheritance with at least one nonsense mutation (29). The clinical features of congenital Ca_V_1.1-related myopathy includes severe generalized muscle weakness and atrophy; in case with a late disease onset – proximal leg weakness with signs of vacuolar myopathy on the muscle biopsy (29, 30). Our patient had not any clinical signs of myopathy, also creatine kinase level was normal and needle EMG did not show myogenic changes.

Another disease caused by *CACNA1S* gene mutations is NormoKPP. We were unable to exclude the diagnosis of NormoKPP because the blood potassium level was never examined during the attack; however, in all previously published cases with mutations in the R900 position the diagnosis of HypoKPP was laboratory confirmed (9, 10). Also the positive effect of the potassium supplementation and spironolactone indirectly confirms the diagnosis of HypoKPP.

The amplitude of the gating pore current contributing to the development of HypoKPP depends on the number of mutated Arg residues and the size of the side chains of the newly included residues (4, 26). Thor et al. (31) showed the appearance of such current through the Na_V_1.4 channels when R2 in S4 VSD I is mutated to Trp. However, the amplitude of this current was approximately two times less compared to the mutation of the same residue on Gly. In our case, only one of the two outermost arginine residues in S4 VSD III is mutated to Met, which has a rather bulky side chain. Obviously, this mutation will contribute less to the flow of gating pore current compared to the R900G and R900S mutations (see Table 1).

We suppose that multiple pathophysiological mechanisms are involved in the development of HypoKPP in our patient, including depolarization of muscle cells due to leak current through the VSD III, disruption the gating of the main pore of the Ca_V_1.1 channels, and, possibly impaired calcium release from the SR through RYR1. We hypothesize that these pathophysiological mechanisms may be involved to varying degrees in the development of the disease in different patients (see Table 1). In patients with more gentle attacks, depolarization of muscle cells due to leak current may predominate, and in our patient (severe attacks), the latter two causes may contribute more significantly to the development of HypoKPP. Further studies, including molecular dynamics simulations, are needed to provide a better understanding of the extent to which different pathophysiological mechanisms are involved in HypoKPP caused by the IIIS4 R900M mutation.

## Data availability statement

The data presented in the study are deposited in the https://www.ncbi.nlm.nih.gov/clinvar/ repository, accession number VCV002575080.1.

## Ethics statement

The studies involving humans were approved by the Research Center of Neurology Ethical Committee. The studies were conducted in accordance with the local legislation and institutional requirements. The participants provided their written informed consent to participate in this study. Written informed consent was obtained from the individual(s) for the publication of any potentially identifiable images or data included in this article.

## Author contributions

EN: Conceptualization, Data curation, Investigation, Methodology, Writing – original draft, Writing – review & editing, Visualization. AA: Conceptualization, Data curation, Investigation, Methodology, Writing – original draft, Writing – review & editing. AR: Investigation, Methodology, Software, Visualization, Writing – original draft. AP: Investigation, Methodology, Writing – review & editing. NA: Investigation, Methodology, Writing – review & editing. NS: Supervision, Writing – review & editing. SI: Supervision, Writing – review & editing, Project administration.
